# Identification and classification of ATS in oral fluid based on Ag nanoassemblies on Si surface doped with Au nanobipyramids

**DOI:** 10.1038/s41598-023-41860-5

**Published:** 2023-09-04

**Authors:** Sheng-Feng Cui, Hai-Long Yang, Xin Huang, Jing-Wei Wan

**Affiliations:** Center for Traffic Evidence Technology, Zhengzhou Key Laboratory of Criminal Science and Technology, Department of Criminal Science and Technology, Railway Police College, Zhengzhou, 450053 People’s Republic of China

**Keywords:** X-ray diffraction, Bioanalytical chemistry

## Abstract

Herein, a novel Ag NP substrate doped with Au nanobipyramids was designed and fabricated via a convenient procedure of galvanic reaction for the identification and classification of amphetamine-type stimulants (ATS) in oral fluids in combination with surface enhanced Raman scattering (SERS). The substrate was shown to have a three-dimensional nanostructure, high SERS activity, and good stability. In combination with SERS, the Ag NP substrate doped with Au nanobipyramids was able to detect ultra-low traces of ATS, including amphetamine, methylamphetamine (MA), 3,4-methylenedioxyamphetamine (MDA), and 3,4-methylenedioxymethylamphetamine (MDMA) in oral fluid with limit of detection (LOD) and limit of determination quantitation (LOQ) as low as 10^–9^ mg/mL, which is much better than the current spectroscopic techniques. The equations between concentration and peaks intensity for quantitative analysis displied good doublelogarithmic linear relations and reliability figures of merit at nanogram concentration level in compartion with GC–MS method. The approach can be broadly applied to the ultra-low trace detection of ATS in oral fluid and would be particularly useful for the analyses of nitrogenous organic compounds.

## Introduction

Over the past decade, misuse of amphetamine-type stimulants has grown significantly worldwide. The high prevalence and associated morbidity and mortality of ATS abuse is now a major public health issue, with a sharp increase in overdose deaths related to ATS^1^. According to the Substance Abuse and Mental Health Services Administration, the number of people over the age of 12 misusing ATS increased from 1.7 million in 2016 to 5.1 million wordwide in 2020. Therefore, there is a growing need to the control ATS abuse^2^. The identification and classification of ATS involve a crucial step in the process of fighting against the crime of drug production, trafficking, and abuse, with ATS detection in biological specimens playing a significant role in drug screening, including of addiction, drugged driving, and monitoring of regional drug abuse^3, 4^.

Currently, oral fluid is widely accepted as an alternative specimen to urine and blood for testing of illicit drugs because it allows measurement of drug concentration levels and is easy to collect by non-medical personnel^5, 6^. Crime laboratories rely on a variety of methods to detect suspected ATS in oral fluid, including Au immunochromatographic assays, chromatography (thin-layer chromatography, liquid chromatography, and high-performance liquid chromatography), and chromatography-mass spectrometry^7^. Nevertheless, despite advances in the field, these chromatography techniques require significant time investment, human resources, and expertise as well as harmful organic solvents. Furthermore, immunological testing methods have a considerable probability of yielding false positive or false negative results^8^. Other challenges include the required sensitivity for screening to avoid true positive and false positive responses, large sample amounts needed for analysis, and the inherent attributes of various molecules. Therefore, an oral fluid-based, portable, completely automated, miniaturized, and one-touch device for illicit drug abuse screening is required.

SERS is a highly surface-sensitive and selective technique that enhances Raman scattering by molecules adsorbed on rough metal surfaces or by nanostructures such as plasmonic-magnetic silica nanotubes, Au, and/or Ag nanoparticles (NPs)^9, 10^. The enhanced Raman signal arises from the electromagnetic interaction of light with the metals, known as localized surface plasmon resonance^11^. The surface plasmon resonance effect is determined by the shape, size, and dielectric constants of both the metallic NPs and the surrounding material^12^. As the shape or size of NPs changes, the surface geometry changes, leading to a shift in surface electric field density. To enhance the Raman signal, the sample molecules must be adsorbed on or very close to the surface of the metal NPs. At a maximum distance between the sample and metal surface of ~ 10 nm, the SERS signal can achieve more than 8 orders of magnitude greater scattering than Raman signal. Consequently, SERS is among the most sensitive analytical techniques for the detection of low concentrations (nanogram level) of a wide range of compounds in different matrices^13, 14^.

The identification and classification of illicit ATS in oral fluid is an issue of primary importance for forensic laboratories and practical work. To the best of our knowledge, few studies have been reported on the detection of ATS in oral fluid with SERS. Previous methods based on Au-doped and Ag-doped sol–gel substrates need a complex system of capillaries and sample pretreatment, and their reproducibility is poor. Herein, we explore a new procedure for the identification and classification of ATS in oral fluid samples with SERS specificity by employing an Si substrate coated with Ag NPs doped with trace amounts of Au nanobipyramids. The distance between ATS molecules and the metal surface was achieved through electrostatic interactions between organic cations. The results herein will provide rapid, confirmatory, and non-destructive analyses to detect ultra-traces of ATS in oral fluid and achieve a very low LOD and LOQ while using a relatively simple analytical approach. This approach will have practical application in the field of forensic science.

## Experimental section

### Reagents and samples

Amphetamine, MA, MDA, and MDMA (standard substance, 1 mg/mL in methanol solution), hexadecyltrimethylammonium bromide (CTAB), and hydrofluoric acid (49% in H_2_O, 99.99% metals basis) were purchased from Sigma-Aldrich (St. Louis, MO, USA). H_2_SO_4_ (98.08%), H_2_O_2_ (30%), HCl, and AgNO_3_ were obtained from Hengxing (Tianjin, China); DP-type (100) 4″ Si wafers were purchased from Lijing Optoelctronics (Suzhou, China). Au nanobipyramids (0.1 mg/mL in H_2_O, absorption peak 785 nm, 20 nm) were obtained from Zhongkeleming (Beijing, China). The oral fluid samples were donated by non-consumer volunteers, and informed consent was obtained from all subjects. Ultrapure water (18.25 MΩ/cm) was prepared using a Milli-Q Advantage ultrapure water system ((Millipore, Germany). All chemicals were used without further treatment. CTAB, AgNO_3_, and HF were dissolved in ultrapure water to prepare aqueous solutions (CTAB, 4.2 × 10^–3^ mol/L; AgNO_3_, 0.03 mol/L; and HF, 0.75 mol/L). All the methods in our work were carried out in accordance with relevant guidelines and regulations, and the experimental protocols were approved by Railway Police College Ethic Committee.

### Instrumentation and signal acquisition

All Raman spectra were acquired with the Thermo Fisher DXR2xi Raman microscope employing a 10 × objective lens and excited at 785 nm. Spectra were obtained in triplicate for each sample, with an exposure time of 25 s for each spectrum (10 mW laser power and a 50-μm slit aperture), and collected by Raman Soft 1.04 software. The spectral region was set from 600 to 1800 cm^−1^. No time-dependent SERS vibrational features were observed at the incident laser fluence used in these studies. Each sample was measured five times in parallel, and the spectra were obtained without any baseline correction. Scanning electron microscopy (SEM) images were taken with a FEI Quant FEG 650 (Hillsboro USA) microscope with a field emission gun operating at 30 kV, and the elementary composition of Ag NPs was analyzed (map scanning, scan region: 2 μm × 2 μm) by energy dispersive spectrometry (EDS and EDAX; Philadelphia, USA).

### Ag NP substrate synthesis

Si wafers were cut into small pieces with a diamond glass cutter, cleaned in ultrapure water by ultrasonic cleaning for 10 min, and then immersed in Piranha solution for 15 min to remove organic impurities on the Si surface. The cleaned Si wafers were thoroughly rinsed with ultrapure water and immersed in a solution (20 mL) containing HF, AgNO_3_, CTAB, and Au nanobipyramids (0.3 μL). The reactant concentration and reaction temperature were set according to the reported literature. The morphology of the Ag nanostructures was controlled by tuning the reaction time. The Si wafer was quickly transferred to ultrapure water to stop the reaction after completion, and then thoroughly rinsed with ultrapure water. The Ag NP substrate doped with Au nanobipyramids was obtained and kept in ultrapure water for 3 days, until use for SERS acquisition.

### Sample preparation and data processing

Different volumes of the standard ATS solution were remove the organic solvent using a nitrogen gun and then redissolved in oral fluid for 5 min. The prepared samples of oral fluid were adjusted to the desired pH to a constant volume of 1.0 mL, and then centrifuged at 5000 r/min for 10 min to obtain the supernatant liquid. The Ag NP substrate doped with Au nanobipyramids were immersed in the sample solutions for 5 min. For GM-MS method, the prepared samples of oral fluid (1.0 mL) was centrifuged at a speed of 8000 rpm for 30 min, and collected the supernatant (200 μL) after centrifugation. The obtained solvent was treated 50 μL sodium hydroxide solution (0.1%) and 1 mL borax-hydrochloric acid buffer solution, and mixed well. Subsequently, the solvent was added 3 mL ether, swirled for 1 min, centrifuged at a speed of 2500 rpm for 3 min, and collected the organic phase after centrifugation. The solvent was evaporated at a temperature of 45 °C under reduced pressure. The obtained solid was dissolved with 200 μL methanol, and then liquid supernatant was analyzed (external standard method).

Confirmatory analyses were performed using a Shimadzu GC–MS (2010 PLUS) equipted with a HP-1 column (30 m × 0.25 mm × 0.33 mm). A constant flow of helium at 1 mL/min was used as the carrier gas. The injection was made in split mode with a split ratio of 15:1. The oven temperature was set at 170 °C for 1 min, ramped to 230 °C at 10 °C/min, ramped to 280 °C at 30 °C/min, and then a post run was made at 280 °C for 7 min. The mass spectrometer parameters were as follows: transfer line at 280 °C and ion source at 280 °C. Mass spectral data were collected in the scan mode from 40 to 400 m/z^15^.

## Results and discussion

### Galvanic reaction time and doping with Au nanobipyramids

In the process of cathodic and anodic recation on the Si substrate, Ag^+^ was reduced to Ag to produce Ag nanostructures on the Si surface with the help of CTAB. CTAB molecules undergo two main functions: formation of micelle templates to control the deposition of Ag NPs, followed by absorption onto and coverage of the formed Ag NPs to prevent Ag dendrite growth. The resulting stable bilayer structure of the shell has a positively charged Ag NPs surface surrounded by a large number of electrons. This bilayer capping shell is key to controlling the distance between the metal surface and the molecule to be measured when obtaining the enhanced Raman signal, and is the reason why it is difficult for most small organic molecules with a rich electron surface to adsorb onto the surface of NPs, resulting in failure to obtain Raman signal enhancement^16^. Herein, the galvanic reaction time, Au nanobipyramid doping, and electrostatic interactions were employed to obtain the required distance between the Ag NPs and the molecules.

**Figure 1:**
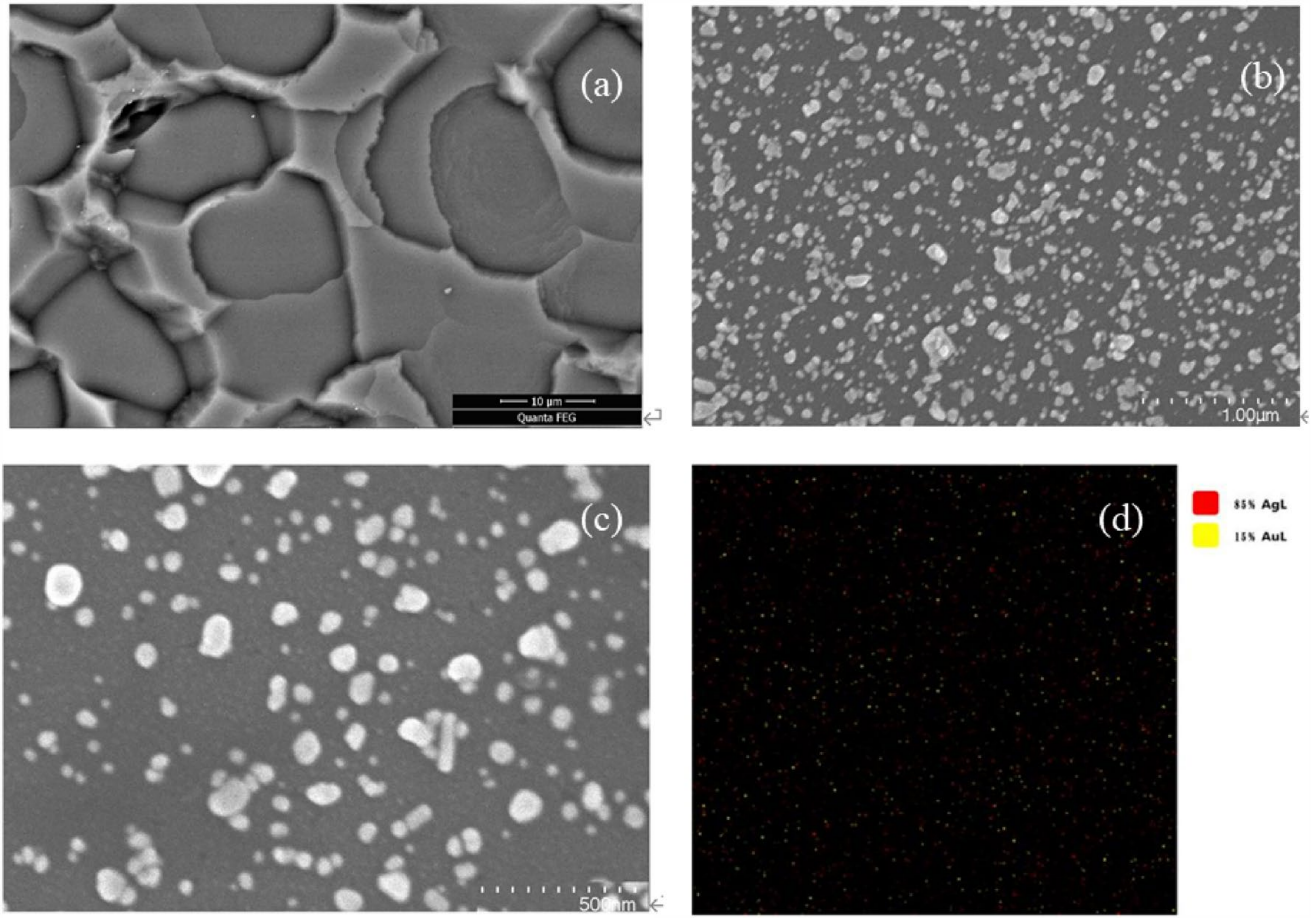
SEM images of obtained Ag NPs. (**a**) ‘rough’ surface of Si substrate at a micro-level (10 μm), (**b**) ‘rough’ surface of Si substrate at a nano-level (1 μm), (**c**) ‘rough’ surface of Si substrate at a nano-level (500 nm), (**d**) spectra of SEM/EDS of nanoparticles (map scanning, scanning region 2 μm × 2 μm).

The ATS molecule was shown to be adsorbed on the ‘rough’ surface of the processed Si wafers, producing an enhanced surface Raman signal (Fig. 1a). Conversely, an ATS molecule Raman signal was not observed on the ‘smooth’ surface of the processed Si wafers. On average, ~ 65 Ag NPs were formed per 1 μm^2^ of Si substrate surface (Fig. 1b,c). The NP size ranged from 20 to 32 nm, accounting for 68% of the total surface area. SEM/EDS showed that ~ 85% of the NPs displayed Ag (L) characteristic peaks, and 15% percent of the NPs showed Au (L) characteristic peaks in EDS (Fig. 1d), suggesting that the Au nanobipyramids were wrapped by Ag NPs on the Si substrate. Ag NPs doped with Au nanobipyramids exhibited a 3D nanostructure with a high density of nanotips and deep nanogaps.

**Figure 2:**
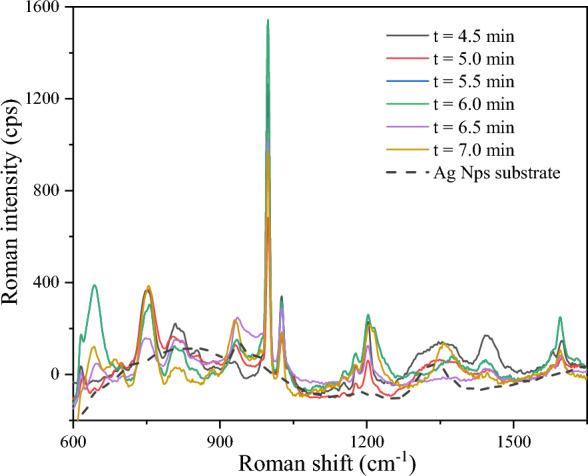
SERS of MA (1.0 × 10^−6^ mol/L) on the Ag NPs substrates prepared at different reaction times.

Although the required reaction time to obtain an enhanced Raman signal has been discussed in previous studies, herein, the galvanic reaction time varied for different compounds and exerted considerable influence on the SERS of ATS (Fig. 2). The size and shape of the formed Ag nanostructures dramatically changed with the reaction time, and the doping Au nanobipyramids further affected the formed Ag nanostructures. At 6.0 min of reaction time and using 1 μL of Au nanobipyramids in a constant volume of 20.0 mL, the Raman intensity reached a maximum, demonstrating that a high density of high-quality Ag NPs suitable for adsorption of ATS was uniformly distributed on the Si surface.

### Effect of pH

**Figure 3:**
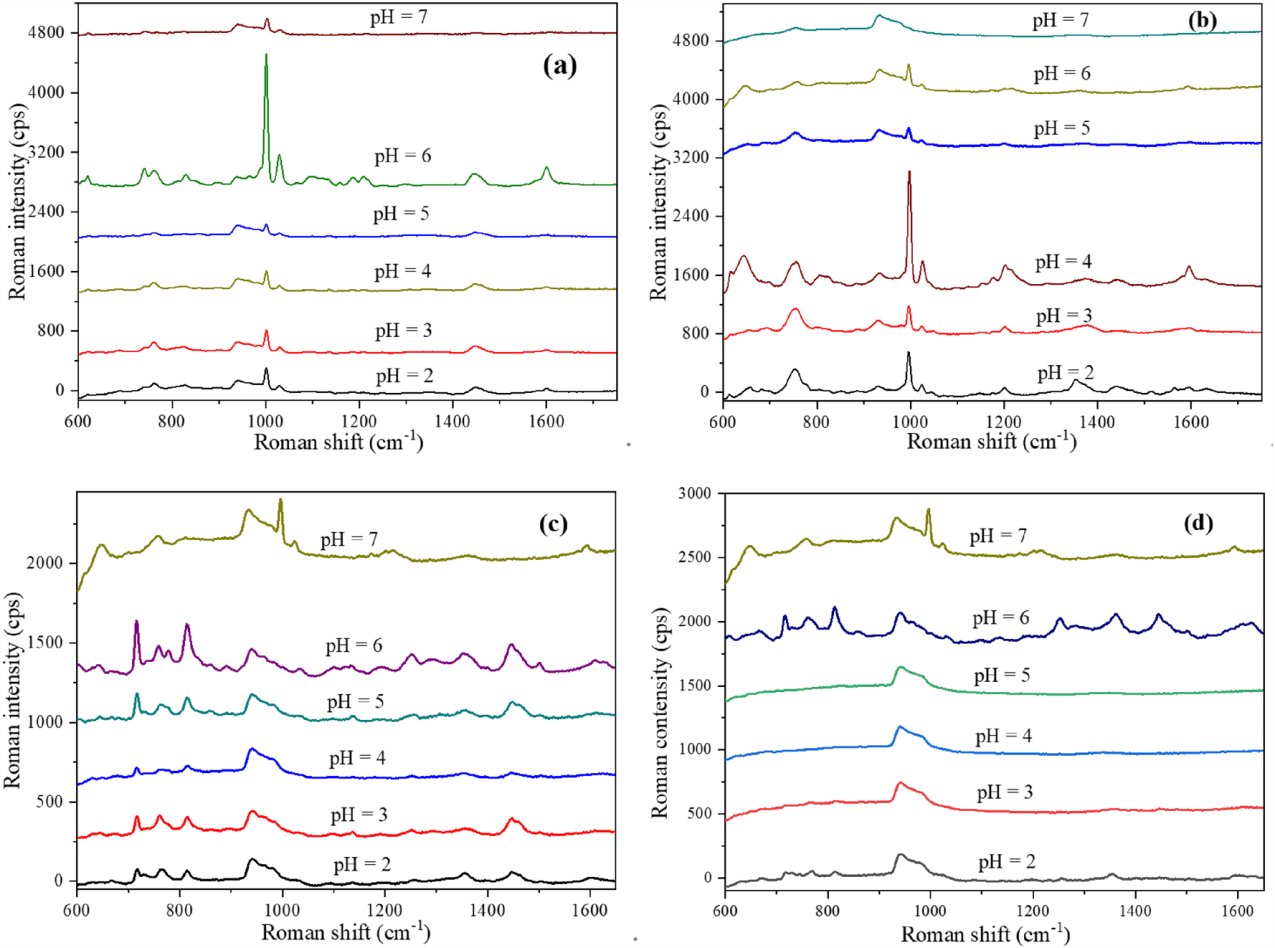
The effect of pH of aqueous solutions on surface-enhanced Raman signal of ATS with same concentrations. (**a**) Amphetamine, (**b**) MA, (**c**) MDA, (**d**) MDMA.

ATS are organic bases with an amine group and are therefore proton acceptors that can be easily protonated. Herein, amphetamine, MA, MDA, and MDMA were protonated by adjusting the pH of the solution to form positive ions. The electrostatic interactions of the amine cation and electrons distributed around the Ag NP bilayer capping shell led to the required distance between the Ag NPs and the molecules. Thus, the protonation of the amino group had a direct effect on the enhanced Raman signal (Fig. 3). At a system pH ≥ 7, amphetamine, MA, MDA, and MDMA exist in a form of organic molecules, and showed a very weak Raman signal because they were not adsorbed on the Ag NP substrate. As a contrast, other commercialized Au/Ag NPs substrates were purchased for the same study, and the results showed the commercialized Au/Ag NPs substrates cannot obtain enough enhanced Raman signals regardless of the pH of the solution.

Under the condition of amino protonation, electrostatic interaction between protonated ATS molecules and Ag NPs was the leading driving force to meet distance requirements, thus leading to successful the SERS of ATS. However, the enhancement of Raman signals depends on the degree of ATS protonation. The pH values of the various ATS compounds differ slightly due to their different pKa values (Fig. 2). Based on the physicochemical properties of ATS, the amine group is completely protonated in a solution at pH 5, showing optimal enhanced Raman signal. The optimal pH value for amphetamine, MDA, and MDMA is 6 to obtain high quality Raman spectra. On the other hand, when the solution is too acidic (pH ≤ 4), the Raman signal is weakened, with some characteristic ATS peaks not appearing in the spectra. These results would indicate that the large number of protons in the solution occupy the space for Ag NPs on the substrate.

### Reliability of the Ag NP substrates

**Figure 4:**
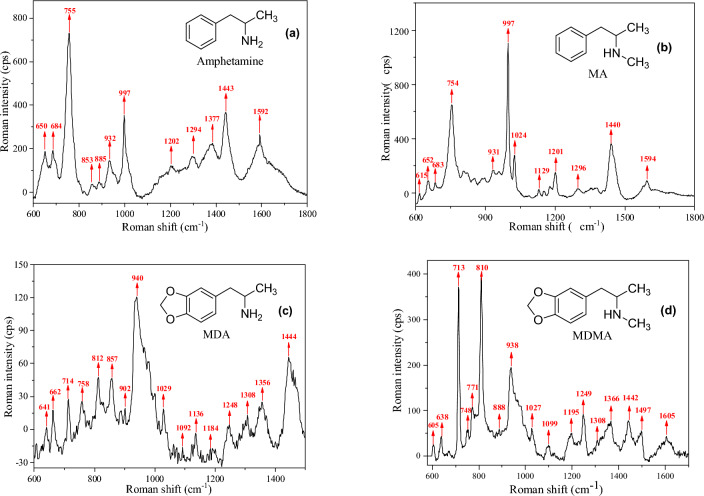
SERS spectra of aqueous solutions of ATS at the same concentration. (**a**) Amphetamine, (**b**) MA, (**c**) MDA, (**d**) MDMA. Each data point were obtained from 5 repeated experiments.

SERS spectra of oral fluid containing ATS were obtained (Fig. 4). Due to their similar chemical structure and major functional groups, amphetamine, MA, MDA, and MDMA have similar characteristic peaks on the Raman spectrum. The characteristic Raman peak of all ATS appeared clearly on the prepared substrate at a very low concentration and the reverse was true at high concentrations. Most of the significant bands at ~ 1592, 1443, 1202, 997, 932, 755, 684, and 650 cm^−1^ were identified as characteristic of amphetamine and MA due to their extremely similar molecular structure. Peaks characteristic of MDA and MDMA were also observed in similar positions. The Raman spectra of the group of amphetamine and MA showed obvious differences with the group of MDA and MDMA, and the Raman spectra peaks of group of MDA and MDMA were more complex. Peaks at ~ 1592 and 997 cm^−1^ correspond to the symmetric modes of aromatic C–C stretching vibrations, those at ~ 1443 and 1202 cm^–1^ were attributed to H–N^+^ symmetric deformation vibration mode and H–N^+^ stretching mode, respectively, and the peak at ~ 754 cm^−1^ was assigned to the HC–N^+^ wagging vibration mode. The substituents in the benzene ring of MDA and MDMA resulted in considerably different SERS spectra. Electron-withdrawing substituents changed the SERS peaks of aromatic C–C stretching and bending vibrations. The peaks attributed to the symmetric and asymmetric phenyl ring breathing modes appeared clearly in the SERS spectra, whereas the Raman peaks of the amine group were less affected. The symmetric deformation vibration, wagging vibration, and stretching of amine groups appeared at 1442, 1248, and 714 cm^−1^, respectively. Thus, all the characteristic peaks of ATS were clearly shown on the enhanced Raman spectrum.

**Figure 5:**
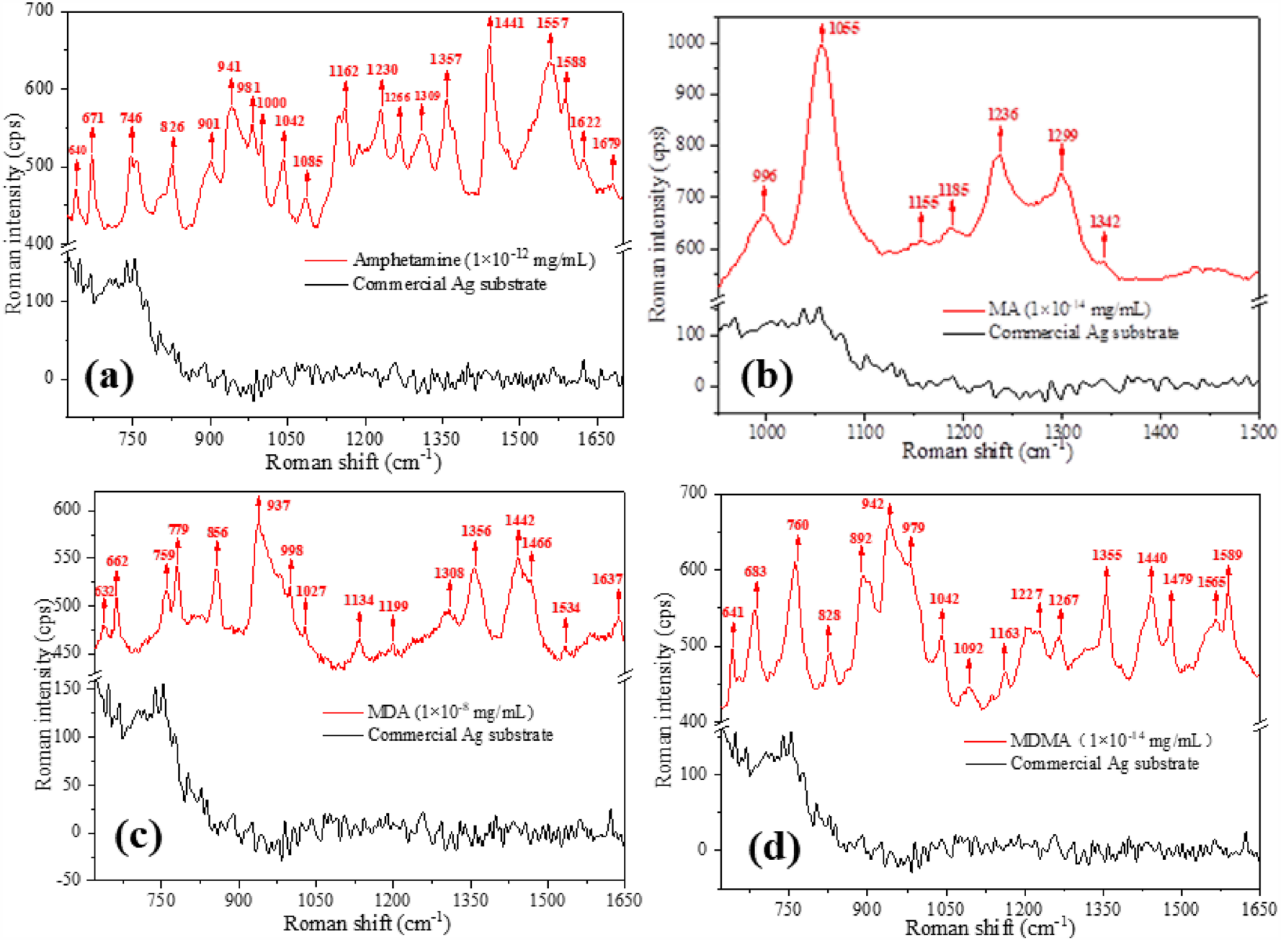
SERS spectra of aqueous solutions of ATS. (**a**) amphetamine (1.0 × 10^–12^ mg/mL), (**b**) MA (1.0 × 10^–14^ mg/mL), (**c**) MDA (1.0 × 10^–8^ mg/mL), (**d**) MDMA (1.0 × 10^–14^ mg/mL). Each data point were obtained from 5 repeated experiments.

To validate the detection capability and stability of the prepared SERS substrate, SERS of ATS solutions at different concentrations and at fixed pH values were obtained, and the commercial Ag NP substrate was also studied as a control. The results showed that the Ag NP substrate produced high-quality SERS spectra even at nanogram ATS concentrations (Fig. 5), and the signal intensity of the ATS characteristic peaks was relatively consistent. Conversely, the commercial Ag NP substrate showed very poor results (black lines in Fig. 5). These results illustrate how adjusting the pH of the solution can lead to different characteristic peaks of ATS components in a SERS spectrum from multi-component oral fluid, and this strategy can be exploited to enhance the identification and classification of ATS based on such spectroscopic signatures.

### Quantitative analysis of ATS

To further study the ability of quantitative analysis of ATS based on the Ag NP substrate, the intensity of characteristic peaks of ATS was measured after immersion in oral fluid solutions at different ATS concentrations. The concentrations of these ATS were chosen based on their existence in human oral fluid in the range of 10^–9^ mg/mL^17^. The characteristic bands at 997 and 755 cm^−1^ were used to estimate concentrations of amphetamine and MA, and those at 970 and 1442 cm^−1^ were employed to estimate the concentrations of MDA and MDMA. The figures of merit for the quantitative method including LOD, LOQ, root mean-square error of prediction (RMSEP) and average recovery (AR) were evaluated using the definitions from IUPAC, and the results were listed in Table 1. A linear relation can be used to quantitatively describe the spectral dependence on ATS concentration

$$\log I = {\text{A}}\log C + B$$

where A and B are the constants independent of the ATS concentration (*C*) in solutions. *I* is the intensity of characteristic peaks of ATS.

**Table 1:** Figures of merit obtained from the quantitative method.

Statistic parameters	Amphetamine	MA	MDA	MDMA
AR (%)	98.3 ± 5.2	101.0 ± 6.1	93.7 ± 3.7	97.9 ± 2.9
RMSEP (mg/mL, 10^–9^)	0.20	0.20	0.32	0.14
LOD (mg/mL, 10^–9^)	22.6	8.7	5.8	11.0
LOQ (mg/mL, 10^–9^)	67.8	27.1	17.4	33.0

**Figure 6:**
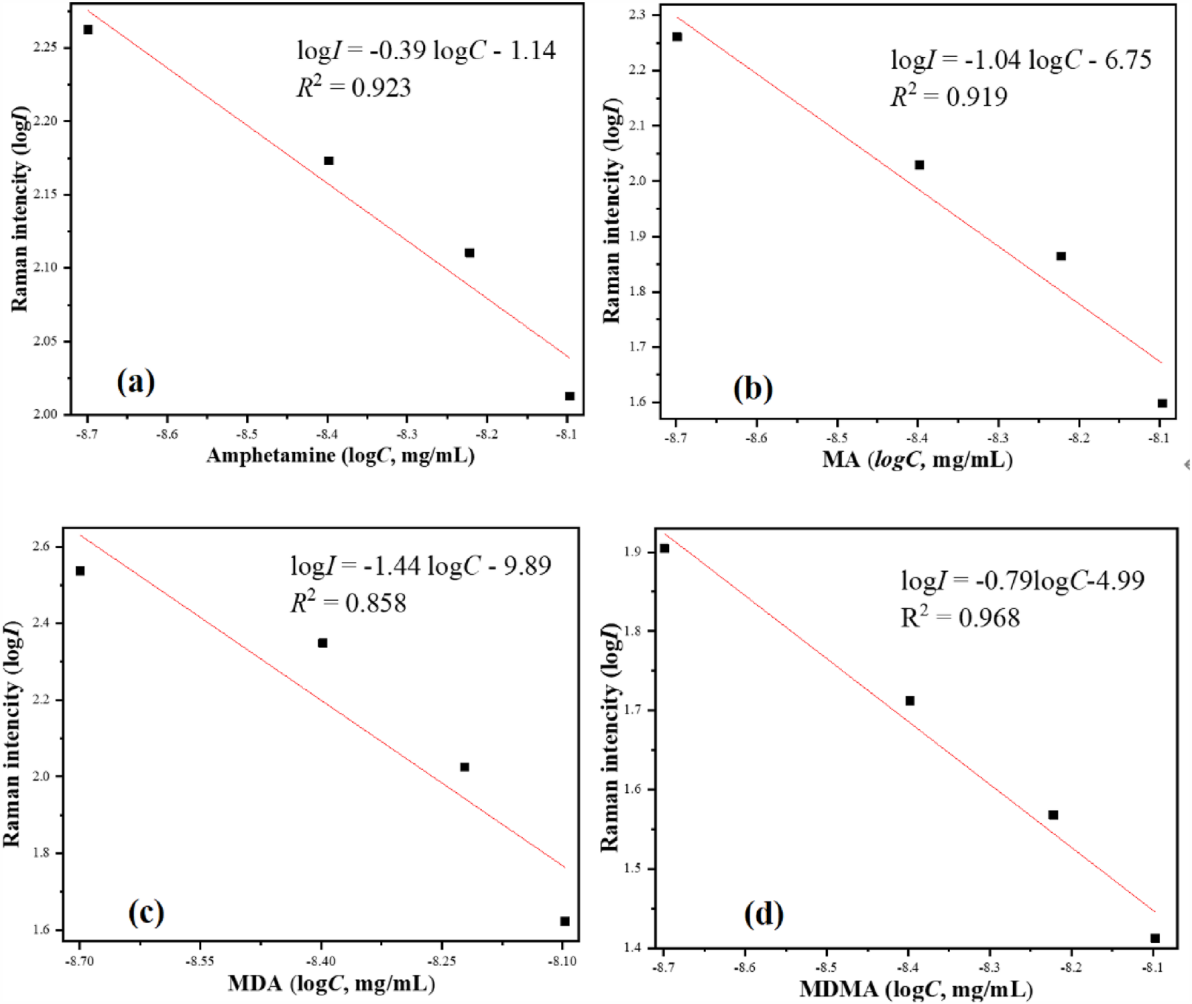
Linear regression equation of peak intensity at 997, 755, 970 and 1442 cm^–1^ versus the concentrations of amphetamine, MA, MDA, and MDMA, respectively at the nanogram (10^–9^ mg/mL) concentration range. (**a**) amphetamine, (**b**) MA, (**c**) MDA, (**d**) MDMA. R is correlation coefficient.

As shown in Fig. 6, the peak intensity (*I*) and concentration (*C*) of ATS show a good doublelogarithmic linear relation in the range of 10^–9^ mg/mL, and the relationship is negative and linear over the range. The values of constants A and B are estimated by linear fitting, and the equation of peak intensity and concentration of ATS are established in the range of 10^–9^ mg/mL, respectively. As shown in Table 1, the low RMSEP (0.14–0.32, mg/mL, 10^–9^) and high recovery value (rangeing from 93.7 to 101.0%) indicated good performance of the SERS method using Ag NP substrate doped with Au nanobipyramids for determination of amphetamine, MA, MAM, and MDMA. The most important results include LODs (5.8–22.6, 10^–9^ mg/mL) and LOQs (17.4–67.8, 10^–9^ mg/mL) for the method that permits to correctly detect amphetamine, MA, MDA, and MDMA in oral fluid at nanogram level.

We futher prepared some prediction samples, and quantitatively detected ATS in oral fluid solutions according to the obtained equation. Chromatographic method (GC–MS) was empolied as Confirmatory analyses. Satisfactory validation outcomes were achieved for the prediction samples of ATS (as shown in Table 2). The prediction results of SERS method using Ag NP substrate doped with Au nanobipyramids have a good correlation with the that of GC–MS. Thus, the Ag NP substrate doped with Au nanobipyramids could be used to achieve the quantitative detection of ATS in oral fluid according to these linear regression equations.

**Table 2:** Predicted concentrations of amphetamine, MA, MDA, and MDMA (n = 5, *p* < 0.01).

Samples	Amphetamine (mg/mL, 10^–9^)		MA (mg/mL, 10^–9^)		MDA (mg/mL, 10^–9^)		MDMA (mg/mL, 10^–9^)	
	SERS	GC/MS	SERS	GC/MS	SERS	GC/MS	SERS	GC/MS
1	1.3 ± 0.10	1.6	1.2 ± 0.05	1.6	1.2 ± 0.08	1.5	1.4 ± 0.10	1.4
2	2.3 ± 0.2	2.5	2.1 ± 0.08	2.3	2 ± 0.1	2.1	2.1 ± 0.11	2.2
3	3.1 ± 0.05	3.3	3.6 ± 0.10	3.4	3.2 ± 0.19	3.2	3.2 ± 0.24	3.6
4	5.5 ± 0.09	5.7	5.8 ± 0.12	5.7	5.4 ± 0.23	5.7	5.5 ± 0.18	5.7
5	7.3 ± 0.12	7.8	7.7 ± 0.23	7.4	7.0 ± 0.3	7.7	7.6 ± 0.25	7.4
6	8.7 ± 0.24	8.6	8.6 ± 0.18	8.5	8.1 ± 0.27	8.6	8.3 ± 0.30	8.6

## Conclusion

Herein, a novel Ag NP SERS substrate doped with Au nanobipyramids was designed and fabricated via a convenient galvanic reaction procedure and was validated to identify and classify ATS components in multi-component oral fluid by adjusting the pH of the solution. Doping with Au nanobipyramids and the electrostatic interactions were successfully optimized to tune the plasmon resonance and absorbance of Ag NPs to match the excitation wavelength in the process of obtaining Raman enhanced signal. SEM/EDS showed that Ag NP SERS substrates had a 3D nanostructure with high structural consistency, high nanotip densities, and deep nanogaps. Based on the charged bilayer capping shell of Ag NPs, the distance between ATS molecule and Ag NPs to meet the resonance requirements was achieved by electrostatic interactions. The fabricated Ag NP SERS substrates have high sensitivity and stability for the detection of trace amounts of ATS, including amphetamine MA, MDA, and MDMA, in oral fluid. The values of LOD and LOQ were as low as 10^–9^ mg/mL, which is much better than the currently available spectroscopic techniques. The equations for quantitative analysis of ATS displied good doublelogarithmic linear relations and reliability figures of merit at nanogram concentration level in compartion with GC–MS method. Our fabricated Ag NP SERS substrate and the strategies for obtaining enhanced Raman signals are of importance in the detection of trace ATS in forensic science. Furthermore, the Ag NP SERS substrate is currently being investigated for the analysis of other types of drugs such as opioids, heroin, and cannabis.

## Data Availability

The datasets used and/or analysed during the current study available from the corresponding author on reasonable request.
